# Association of autoimmune diseases with celiac disease and its risk factors

**DOI:** 10.12669/pjms.35.6.821

**Published:** 2019

**Authors:** Yusuf Kayar, Ramazan Dertli

**Affiliations:** 1 Yusuf Kayar, Department of Internal Diseases, Division of Gastroenterology, Van Education and Research Hospital, Van, Turkey; 2 Ramazan Dertli, Department of Internal Diseases, Division of Gastroenterology, Van Education and Research Hospital, Van, Turkey

**Keywords:** Autoimmune Diseases, Celiac Disease, Risk Factors

## Abstract

**Objectives:**

Autoimmune Diseases (AIDs) are detected in celiac patients. Our purpose was to determine the AIDs that are associated with celiac disease and related risk factors in the Turkish population.

**Methods:**

The study included 230 celiac patients who were diagnosed and followed-up in our clinics between 2015-2019. All AIDs that accompanied the celiac disease were recorded, and their association with risk factors was analyzed.

**Results:**

The mean age of the patients was 35.6±10.6 years (age range:18-72). A total of 58(25.2%) patients were male and the mean age at onset of disease was 29.1±6.5 years. The duration of disease follow-up was 6.5±4.6 years. One hundred and twenty two (53%) patients were on a strict diet, and 72(31.3%) patients had accompanying AID. Hashimoto thyroiditis was found in 39(17%) patients, asthma in 16(7%) patients as the most common comorbidities. There was a significant relation between AID and female gender, age of diagnosis being <40 years, duration of disease, non-GIS symptoms at the time of admission, and non-employment status.

**Conclusion:**

Screening other AIDs in celiac patients are important, especially in individuals who have risk factors. Considering that many AIDs may develop in the future despite dietary compliance, patients should be followed with a multidisciplinary approach.

## INTRODUCTION

Celiac Disease (CD) is an autoimmune enteropathy based on gluten intolerance in genetically predisposed individuals, and is characterized by inflammatory process resulting in flattening of the villus, which occur in the small intestine, and loss of absorption function.^1^,^2^ As it is the case in other autoimmune diseases (AIDs), the immune response against antigens results in the production of autoantibodies and tissue destruction in CD.^3^ The disease is related closely to genes encoding HLADQ2 and HLADQ8.^3^

It was reported in previous reports that; there is a significant increase in the prevalence of AIDs like Insulin-Dependent Diabetes Mellitus (IDDM), thyroid diseases, connective tissue diseases, skin diseases, autoimmune hepatitis and primary biliary cirrhosis in celiac patients.^4^ This rate was found to be 34% for the first time in the study that was conducted by Ventura et al. with 909 celiac patients, and it was argued that long-term exposure to gluten triggered the development of AIDs.^5^ In this respect, in a study in which 703 patients who had CD were evaluated in Finland, it was determined that the rate of AIDs was 31%; and in a study that was conducted in France, 924 patients were analyzed, and the rate of AIDs was reported as 19.3%.^6^,^7^ It was argued that this might share a pathogenic basis that included the same environmental triggers, genetic disposition, impaired intestinal barrier, and increased permeability in the intestine.^4^,^8^

Although contradictory results were reported in previous studies, it was argued that there is a significant relation between AIDs development and some risk factors in celiac patients.^6^,^7^ In the literature, there are studies showing that; there are significant relations between the presence of AIDs in the family, diagnosis of CD in early decades, duration of gluten exposure, presence of female gender, presence of non-gastrointestinal system (GIS) symptoms, and development of AIDs.^6^,^7^ In some studies that were conducted by considering the development of AIDs as secondary to prolonged gluten exposure, it was shown that serum insulin-related antibody and anti-thyroid antibody levels were reduced at a significant level in the follow-up of children who were fed without gluten; however, in some other studies it was reported that a gluten-free diet did not prevent the development of AIDs.^9^,^10^ In the present study, the purpose was to determine the AIDs accompanying the CD in Turkish population; and to investigate the risk factors affecting the development of AIDs.

## METHODS

### Subjects

The patients who were diagnosed and followed-up in the clinics of Gastroenterology were included in the present study. The celiac patients whose records were included in the hospital database between 2015 and 2019, and newly-diagnosed celiac patients in our outpatient clinics were included in the study. For all patients who were diagnosed with CD, the inclusion criteria for the study were having positive results of the Antibody level test (Anti-Endomysium and Tissue Transglutaminase Antibody (anti-TTG)), which is carried out on clinical and laboratory suspicion, and having consistent results of the tissue samples taken in endoscopy in histopathological examination according to the Marsh classification. The patients who did not continue follow-ups in our clinic, whose data could not be obtained or who had missing data and who were pregnant were not included in the study. A total of 230 patients with CD, whose ages varied between 18 and 72 years, whose data were complete, and who continued their follow-up regularly, were included in the study. Written informed consent forms were received from all participants.

### Evaluation of Immunological, Demographic and Clinic Features

The demographic and anthropometric characteristics (age, gender, onset of disease, duration of disease, presence of CD in family, presence of AIDs in family, smoking and alcohol use, educational status, marital status, Body Mass Index (BMI)) of the patients included in the study were documented. Height (meter) and weight (kg) measurements were made to calculate the BMI of the patients. The BMI was calculated with the following formula: Weight/Height x Height. The waist circumference, hip circumference, waist/hip rates were also documented. In men, 0.9 waist/hip rate was considered to be risk for abdominal obesity and chronic diseases in men, and 0.85 in women.^11^ The symptoms of the participants at admission were documented. All participants were asked to fill in a questionnaire to evaluate the symptoms that were related with the gastrointestinal system. The presence of diarrhea, constipation, reflux, dyspepsia, abdominal pain, nausea, and vomiting were analyzed in patients in the questionnaire.^12^ In addition to the GIS symptoms, the presence of anemia, thyroid diseases, weight loss, growth retardation, infertility, skin lesions, bleeding complaints, bone disorders, and extrahepatic cholestasis were also documented. The antibody levels were evaluated again at the last visit. The patients who had negative antibody levels were accepted to be in immunological remission.

### Dietary Compliance

In patients who had CD, gluten-free diet compliance was questioned retrospectively in the patient records in patient visits by the responsible physician and dietician. In addition, compliance with gluten-free nutrition was evaluated by analyzing the questionnaire that was given to the patients. The diet was classified as two categories; 1) Strict diet (complete dietary compliance), 2) Normal gluten-containing diet and partial dietary compliance.

### Autoimmune Diseases

The presence of AIDs in celiac patients was determined with a structured questionnaire form and patient records (AIDs were diagnosed with anamnesis, physical examination findings, laboratory and imaging methods). The questionnaire was filled by the doctor during the visit of the patient. The presence of AIDs was questioned in all subjects with a predetermined list. IDDM, autoimmune thyroid diseases, autoimmune hepatitis, primary biliary cirrhosis, dermatitis herpetiormis, collagen tissue diseases, immune thrombocytopenia, asthma, autoimmune hypoparathyrodism, autoimmune hypogonadism, and skin diseases were assessed separately. (Dated November 11, 2014) was obtained from the Ethics Committee of our hospital. All the applied procedures were complied with the ethical standards of human testing committee of our institution and the Helsinki Declaration.

### Data Analysis

The results of our study were analyzed with “the Statistical Package for Social Sciences 19.0 (SPSS Armonk, NY: IBM Corp.)” program. The data that had continuous values were given as (mean±standard deviation), and the categorical data were given as frequency and percentage (n, %). The data were tested for compliance to normal distribution with the Kolmogorov-Simirnov Test, Histogram and ± SD. The nonparametric data of the groups were compared by using the Mann Whitney U-test; and the parametric data were compared with the Parametric T-test. The Chi-square Test was employed to test the categorical data. In multivariate analysis, the independent predictors were evaluated by using the Logistic Regression Analysis to predict the AID development related to the CD by using possible factors that were determined in previous analyzes. A P value <0.05 was considered to be statistically significant.

## RESULTS

A total of 230 patients who were followed-up with CD were included in the study. A total of 172(74.8%) of the patients were female, and the mean age was 35.6±10.6 years (range between 18-72 years). The mean age of onset of disease was 29.1±6.5 years (range between 4-66 years). The disease duration was between 1-29 years, and the mean duration was 6.55±4.6 years. The mean BMI level of the patients was 22.1±4.1, waist-hip ratio of the patients ranged between 0.63 and 0.98 with a mean of 0.81±0.07. The waist-hip rate was 0.87±0.06 in males, and 0.8±0.06 in females. In 46(20%) of the patients, it was determined that the waist-hip rate was above normal. According to the March classification, when stage 1, 2 and 3a were evaluated as mild and stage 3b, 3c and stage 4 were evaluated as advanced disease, it was determined that 101(43.9%) patients were mild, and 129(56.1%) patients were in severe stage.

Evaluation for AIDs revealed that; 72(31.3%) of the 230 patients had AIDs. A total of 45(19.6%) of these patients had only one AIDs, 18(7.8%) had two AIDs, and 9(3.9%) had three and more AIDs. Hashimoto thyroiditis was determined in 39(17%) patients, asthma in 16(7%) patients, IDDM in 10(4.3%) patients, dermatitis herpetiformis and autoimmune hepatitis in 8(3.5%) patients each, Sjogren in 6(2.6%) patients, autoimmune hypoparathyrodism in 4(1.7%) patients, immune thrombocytopenia in 3(1.3%) patients, Grave’s disease, primary biliary cirrhosis, psoriasis, Behcet’s disease, scleroderma, dermatomyositis, familial mediterranean fever, and rheumatoid arthritis in 2(0.9%) patients each, systemic lupus erythematosus, gout disease, autoimmune hypogonadism and ankylosing spondylitis in 1 patient (0.45%) each (Table-I).

**Table I:** Accompanying autoimmune diseases in celiac patients.

	N - (%)		N - (%)
Dermatitis Herpetiformis	8 (3.5%)	Rheumatoid arthritis	2 (0.9%)
Type 1 Diabetes Mellitus	10 (4.3%)	Asthma	16 (7%)
Hashimoto Thyroiditis	39 (17%)	Immune Thrombocytopenia	3(1.3%)
Graves’ Disease	2 (0.9%)	Hypogonadism	1 (0.45%)
Primer Biliary Cirrhosis	2 (0.9%)	Sjogren	6 (2.6%)
Autoimmune Hepatitis	8 (3.5%)	Psoriasis	2 (0.9%)
Systemic Lupus Erythematosus	1 (0.45%)	Autoimmune Hypoparathyroidism	4(1.7%)
Scleroderma	2 (0.9%)	Dermatomyositis	2 (0.9%)
Familial Mediterranean Fever	2 (0.9%)	Gout Disease	1 (0.45%)
Behcet’s Disease	2 (0.9%)	Ankylosing Spondylitis	1 (0.45%)

N: Number of patients.

The factors that might affect the development of AIDs in celiac patients were evaluated. There was a statistically significant relation between AIDs development and female gender, age of diagnosis under 40 years, long duration of illness, presence of non-GIS symptoms at the time of first admission and working status. Although the AIDs development was less in patients who had strict diet; however, this was not statistically significant. No significant relations were detected between AID development and other risk factors. While the rate of being young (below 40 years) was 87.5% in individuals with autoimmune disease accompanying CD, this rate was 75.3% in individuals without autoimmune disease accompanying CD (p=0.035). The duration of CD in individuals who had AID accompanying CD was 7.92±5, and the duration of disease in individuals who did not have AID was 5.92±4.3 (p=0.002). Only 70.9% of individuals without CD associated AID were females, however 83.3% of individuals with CD associated AID were females (p=0,044). Only 74.1% of of individuals without CD associated AID were not working, however 87.5% of individuals with CD associated AID were not working (p=0,022). While 20.9% of individuals without CD associated AID had non-GIS symptoms, 34.7% of individuals with CD associated AID had non-GIS symptoms (p=0.025) (Table-II).

**Table II:** Risk factors associated with the presence of autoimmune disease.

Variables	Celiac Disease without Autoimmune Disease	Celiac Disease with Autoimmune Disease	Total	P value
Age (years ± SS)	35.9±12.07	34.9±10.7	35.6±10.6	0.57
Onset age of the disease (years)	30±11.7	27±10.4	29.1±6.5	0.09
Onset age of the disease (years)				
<40 years of age	119 (75.3%)	63 (87.5%)	182 (79.2%)	0.035*
≥40 years of age	39 (24.7%)	9 (12.5%)	48 (20.8%)	
Disease duration (years)	5.92±4.3	7.92±5	6.55±4.6	0.002*
BMI (kg/m^2^)	22±4	22.4±3.9	22.1±4.1	0.49
***Waist/hip rate***				
Below the limit	127 (80.4%)	57 (79.2%)	184 (80%)	0.83
Above the limit	31 (19.6%)	15 (20.8%)	46 (20%)	
***Disease stage***				
Mild (Stage 2-3a)	74 (46.8%)	27(37.5%)	101(43.9%)	0.18
Severe (Stage 3b,3c,4)	84 (53.2%)	45(62.5%)	129 (56.1%)	
***Dietary Compliance***				
Strict	90 (57%)	32 (44.4%)	122 (53.1%)	0.078
None	68 (43%)	40(55.6%)	108 (46.9%)	
***Gender***				
Female	112(70.9%)	60(83.3%)	172 (74.7%)	0.044*
Male	46 (29.1%)	12(16.7%)	58 (25.3%)	
***Immunologic Remission***				
Yes	70 (44.3%)	38 (52.8%)	108 (46.9%)	0.23
No	88 (55.7%)	34 (47.2%)	122 (53.1%)	
***Family History***				
Yes	39 (24.7%)	11 (15.3%)	50 (21.7%)	0.10
No	119 (75.3%)	61(84.7%)	180 (78.3%)	
***Educational Status***				
Illiterate	30 (19%)	16 (22.2%)	46 (20%)	0.38
Primary Education	87(55.1%)	37 (51.4%)	124 (53.9%)	
High School and over	41(25.9%)	19 (26.4%)	60 (26.1%)	
***Marital Status***				
Married	114 (72.2%)	50 (69.4%)	164 (71.3%)	0.67
Single	44 (27.8%)	22 (30.6%)	66 (28.7%)	
***Working Status***				
Working	41 (25.9%)	9 (12.5%)	50 (21.7%)	0.022*
Unemployed	117 (74.1%)	63 (87.5%)	180(78.3%)	
***Smoking Status***				
Smoking	26 (16.5%)	18 (25%)	44 (19.1%)	0.20
Non-smoking	116 (73.4%)	50(69.4%)	166 (72.2%)	
Quit	16 (10.1%)	4(5.6%)	20 (8.7%)	
***Complaints at First Admission***				
GIS symptoms	125 (79.1%)	47 (65.3%)	172 (74.8%)	0.025*
Non-GIS symptoms	33(20.9%)	25 (34.7%)	58 (25.2%)	

BMI: Body Mass Index, GIS: Gastrointestinal System

* Statistically significant (p<0.05)

The logistic regression model that included factors like having GIS symptoms at admission, the age of onset of CD being <40 years, who had a history of CD in the family, having immune remission, gender, working status, having non-GIS symptoms at first admission were used to examine the independent risk factors that determined presence of AIDs accompanied CD. As a result of the analysis, it was determined that the following were independent risk factors in AID development; having non-GIS symptoms at first admission (OR:2.29, 95% CI:1.195-4.388, p=0.013); the age of onset of the disease being <40 years (OR: 2.299, 95% CI:1.030-5.131, p=0.042); and unemployment of the patient (OR: 2.928, 95% CI:1.195-4.388, p=0.013).

## DISCUSSION

In previous studies, it was shown that; there is a significant increase in the prevalence of AIDs in celiac patients and their first-degree relatives when compared to the normal population.^3^ Especially, since type 1 diabetes mellitus and hashimoto’s thyroiditis often accompany CD. For that, routine screening of these AIDs is recommended in all patients with CD.^13^,^14^ Ventura and Viljama conducted a study and showed that the AIDs prevalence was 34% and 31% in celiac patient. They also showed that thyroid diseases, IDDM, asthma and skin diseases accompanied CD frequently.^5^,^6^ Bibbo et al. conducted a study and determined an AID in the control group at a rate of 15.2%. They also showed that this rate was 35.3% in CD patients.^15^ Demirezer Bolat et al. conducted a study with 145 patients who had CD, and reported that 33.1% of the patients had accompanying AIDs to CD; and that the following were the most frequent accompanying diseases: hashimoto thyroiditis (24.1%), type 1 diabetes mellitus (5.5%) and psoriasis (2.8%).^16^ These data show similarities with the findings of our study.

In our study in which 230 celiac patients were included, it was determined that 72 (31.3%) patients had AIDs, and that 39 (17%) patients had hashimoto thyroiditis, 16 (7%) patients had asthma, and 10 (4.3%) patients had type 1 diabetes mellitus as the most common AIDs. It was reported that the pathophysiological basis of the relation between CD and AIDs was sharing of HLA-DR3, HLA-DQ2 and other genetic loci that played roles in AID development.^17^,^18^ In addition, exposure of an immature immune system to gliadin in sensitive individuals causes an immune response modification at an early time, and as a result, an AID together with CD. For these reasons, it is considered that the AID risk is high in celiac patients.^19^

Many environmental factors play roles in the basis of CD and other AID in addition to genetic characteristics. Viljama et al. conducted a study and showed that there was a significant relation between early diagnosis of CD, disease duration, female gender, long duration of gluten exposure, and coexistence of AID.^6^ Similarly, Cosnes et al. conducted another study and found a significant relation between presence of AIDs in family, presence of non-GIS symptoms in patients at admission, and development of AID in patients with diagnosis of CD at age < 36 years.^7^ Bibbo et al. also showed that long disease duration is a predictive factor in AID development.^15^ In the present study of ours, it was found that the age of disease diagnosis being <40 years of age, non-GIS symptoms at first admission, and unemployment of the patient were independent risk factors for AID development.

However, it was observed that the reported data are contradictory in studies examining the relation between gluten-free diet adaptation and AID development. Some studies showed that organ-specific antibodies were more prevalent in celiac patients who were not treated, and gluten exposure time was a predisposing factor in AID development.^5^,^20^ Ventura et al.^5^ showed that organ-specific antibodies were reduced at a significant level in patients who meet gluten-free dietary therapy and that gluten-free diet prevents the development of AID. However, in some previous studies, it was argued that applying gluten-free diet therapy does not prevent AID development; however, in some other studies, it was reported gluten-free diet therapy decreases AID development, yet, it did not provide total protection.^6^,^7^,^10^ Also, Ouaka-Kchaou et al. showed that duration of the disease was longer at a significant level in CD patient accompanied by AID. They also determined that strict dietary compliance was 46% in the AID-accompanied group, and 67% in the group without AID.^21^

In the present study of ours, although it was determined that there were no significant relation between strict dietary compliance and AID development, it was found that diet adaptation was less in the group in which AIDs accompanied CD. In addition, we believe that; the significant relation between prolonged disease duration and AID development suggest that longer exposure to gluten of celiac patients increases the risk of AID development (due to non-compliance to diet).

In Turkey, there are no comprehensive studies that examine the risk factors related to AID and development of the accompanying diseases in a large population. Since the patients who were followed in polyclinics of important healthcare center receiving patients from all surrounding cities were included in our study, a large geographic population was analyzed in the present study. However, there are some limitations of our study. Firstly, although all patients were interviewed face-to-face, and all the data were checked for accuracy, the data were collected retrospectively. Secondly, the patients who did not have clinically obvious complaints; however, who could have subclinical AIDs were not included in the present study. Another limitation of the present study of ours is that; it was not possible to predict the amount of gluten patients took in the long term in an accurate manner. The patients’ remission status was evaluated as clinical laboratory and immunological. However, due to the lack of an objective measurement method for the amount of gluten taken, dietary compliance was determined by using a questionnaire filled with dietician and using the information obtained from the registration data.

## CONCLUSION

In conclusion, our study has shown that; it is important to screen other AIDs in celiac patients, especially in those who have risk factors. It must be kept in mind that patients who present with GIS symptoms must be screened in terms of CD, and also, patients who present with non-GIS complaints may have CD, and that AIDs accompanies CD more in patients presenting with these complaints. Because of the presence of many AIDs and extra-intestinal involvements in celiac patients, these patients must be treated in an integrity with a multidisciplinary approach.

## References

[1] Rubio-Tapia A, Hill ID, Kelly CP, Calderwood AH, Murray JA (2013). ACG clinical guidelines:diagnosis and management of celiac disease. Am J Gastroenterol.

[2] Di Sabatino A, Corazza GR (2009). Coeliac disease. Lancet.

[3] Zanoni G, Navone R, Lunardi C, Tridente G, Bason C, Sivori S (2006). In celiac disease, a subset of autoantibodies against transglutaminase binds toll-like receptor 4 and induces activation of monocytes. PLoS Med.

[4] Alaedini A, Green HR (2005). Narrative Review:Celiac Disease:Understanding a Complex Autoimmune Disorder. Ann Intern Med.

[5] Ventura A, Magazzù G, Greco L (1999). Duration of exposure to gluten and risk for autoimmune disorders in patients with celiac disease. Gastroenterology.

[6] Viljamaa M, Kaukinen K, Huhtala H, Kyrönpalo S, Rasmussen M, Collin P (2005). Coeliac disease, autoimmune diseases and gluten exposure. Scand J Gastroenterol.

[7] Cosnes J, Cellier C, Viola S, Colombel JF, Michaud L, Sarles J (2008). Incidence of autoimmune diseases in celiac disease:protective effect of the gluten-free diet. Clin Gastroenterol Hepatol.

[8] Sollid LM, Jabri B (2005). Is celiac disease an autoimmune disorder?. Curr Opin Immunol.

[9] Ventura A, Neri E, Ughi C, Leopaldi A, Citta A, Not T (2000). Gluten-dependent diabetes-related and thyroid-related autoantibodies in patients with celiac disease. J Pediatr.

[10] Sategna Guidetti C, Solerio E, Scaglione N, Aimo G, Mengozzi G (2001). Duration of gluten exposure in adult coeliac disease does not correlate with the risk of autoimmune disorders. Gut.

[11] American Diabetes Association (2015). Standards of Medical Care in Diabetes. Diabetes Care.

[12] Revicki DA, Wood M, Wiklund I, Crawley J (1998). Reliability and validity of the Gastrointestinal Symptom Rating Scale in patients with gastroesophageal reflux disease. Qual Life Res.

[13] Ludvigsson JF, Bai JC, Biagi F, Card TR, Ciacci C, Ciclitira PJ (2014). Diagnosis and management of adult coeliac disease:guidelines from the British Society of Gastroenterology. Gut.

[14] Rubio-Tapia A, Hill ID, Kelly CP, Calderwood AH, Murray JA, American College of G (2013). ACG clinical guidelines:diagnosis and management of celiac disease. Am J Gastroenterol.

[15] Bibbò S, Pes GM, Usai-Satta P, Salis R, Soro S, Quarta Colosso BM (2017). Chronic autoimmune disorders are increased in coeliac disease:A case-control study. Medicine (Baltimore).

[16] Demirezer Bolat A, Akin FE, Tahtaci M, Tayfur Yürekli Ö, Köseoğlu H, Erten Ş (2015). Risk factors for polyautoimmunity among patients with celiac disease:a cross-sectional survey. Digestion.

[17] Smyth DJ, Plagnol V, Walker NM, Cooper JD, Downes K, Yang JH (2008). Shared and distinct genetic variants in type 1 diabetes and celiac disease. N Engl J Med.

[18] Fallahi P, Ferrari SM, Ruffilli I, Elia G, Biricotti M, Vita R (2016). The association of otherautoimmune diseases in patients with autoimmune thyroidiitis:review ofthe literature and report of a large series of patients. Autoimmun Rev.

[19] Ventura A, Magazù G, Gerarduzzi T, Greco L (2002). Coeliac disease and the risk of autoimmune disorders. Gut.

[20] Toscano V, Conti F.G, Anastasi E, Mariani P, Tiberti C, Poggi M (2000). Importance of gluten in the induction of endocrine autoantibodies and organ dysfunction in adolescent celiac patients. Am J Gastroenterol.

[21] Ouaka-Kchaou A, Ennaifer R, Elloumi H, Gargouri D, Hefaiedh R, Kochlef A (2008). Autoimmune diseases in coeliac disease:effect of gluten exposure. Therap Adv Gastroenterol.

